# Idelalisib may have the potential to increase radiotherapy side effects

**DOI:** 10.1186/s13014-017-0827-7

**Published:** 2017-06-28

**Authors:** Thomas Gryc, Florian Putz, Nicole Goerig, Sonia Ziegler, Rainer Fietkau, Luitpold V. Distel, Barbara Schuster

**Affiliations:** Department of Radiation Oncology, Universitätsklinikum Erlangen, Friedrich-Alexander-Universität Erlangen-Nürnberg, Universitätsstraße 27, D-91054 Erlangen, Germany

**Keywords:** Idelalisib, Apoptosis, Side effects, Radiotherapy, Kinase inhibitor

## Abstract

**Introduction:**

Idelalisib is approved for the treatment of relapsed chronic lymphocytic leukemia together with Rituximab and for monotherapy of follicular B-cell non-Hodgkin’s lymphoma and small lymphocytic lymphoma. It is a potent and selective phosphatidylinositol 3-kinase-δ (PI3K-δ) inhibitor. PI3K-δ primarily is expressed in B-cells and prevents effectively proliferation in malignant B-cells.

**Methods:**

We provide a detailed report on treatment history and photo documentation of acute adverse effects of radiation therapy with simultaneous Idelalisib medication in one case of B-CLL. Radiosensitivity tests were performed for the index patient under Idelalisib and after the addition of Idelalisib to healthy individuals’ blood. Radiosensitivity in human lymphocytes was analyzed with a three color in situ hybridization assay. Primary skin fibroblasts were studied after a treatment with Idelalisib for apoptosis, necrosis and cell cycle using flow cytometry. DNA double-strand break repair was analyzed by γH2AX immunostaining.

**Results:**

The index patient presented a strong grade 2 radiodermatitis and grade 3 mucositis after irradiation with 20 Gy and a simultaneous intake of Idelalisib. Irradiations without Idelalisib medication were well tolerated and resulted in not more than grade 1 radiodermatitis. The index patient under Idelalisib had a radiosensitivity of 0.62 B/M which is in the range of clearly radiosensitive patients. A combined treatment of lymphocytes with 2 Gy and 10 nmol/l Idelalisib showed a tendency to an increased radiosensitivity. We found a clear increase of apoptosis as a result of the combined treatment in the Idelalisib dose range of 1 to 100 nmol/l compared to solely irradiated cells or solely Idelalisib treated cells (*p* = 0.05).

**Conclusion:**

A combined Idelalisib radiotherapy treatment has an increased risk of side effects. However, combined therapy seems to be feasible when patients are monitored closely.

## Introduction

Idelalisib is a potent and selective phosphatidylinositol 3-kinase-δ (PI3K-δ) inhibitor. It is approved for the treatment of relapsed chronic lymphocytic leukemia together with Rituximab and for the monotherapy of follicular B-cell non-Hodgkin’s lymphoma and small lymphocytic lymphoma [1]. PI3K-δ is essential for antigen-induced B-cell receptor (BCR) signaling. The PI3K-δ pathway regulates cellular growth, proliferation and survival in response to cellular stress, furthermore it plays an important role in many different pathways of B-cell biology. Therefore, the B-cell receptor signaling is a pivotal pathway in CLL pathogenesis and disease progression [1–3]. Several kinases in the BCR pathway can be targeted with small molecules to effectively interrupt BCR signaling in vivo, resulting in the inhibition of activation, proliferationand survival of the tumor cells [4]. Here Idelalisib is one of the most advanced inhibitors of PI3K-δ. Preclinical studies have shown that inhibition of PI3K-δ dependent signaling with Idelalisib resulted in decreased downstream signaling of BCR, CXCR4 and CXCR5 (i.e., decreased activation of AKT, mTOR and other downstream effectors) [5]. Idelalisib therefore deeply interacts with cell physiology, thus resulting in multiple, and various adverse reactions ranging from severe colitis, hepatotoxicity, pneumonitis to severe cutaneous reactions, anaphylaxis, neutropenia and embryo-fetal toxicity. In this study we investigated the interaction of Idelalisib with normal tissue cells and the first case published so far known of radiosensitization suffering from an overshooting skin reaction provoked by a combined radiotherapy and Idelalisib treatment.

## Methods

### Cell culture

SBL5 are primary skin fibroblasts derived from a healthy 20-year-old Caucasian male. Fibroblasts were grown in Ham’s F-12Medium (Biochrom, Berlin, Germany) supplemented with 20% fetal bovine serum, 3 mmol/l L-glutamine and 1% penicillin/streptomycin. Lymphocytes were derived ex vivo from a healthy 29-year old Caucasian female and were short time cultured in RPMI supplemented with 10% fetal bovine serum. All cells were cultured at 37 °C in a 5% CO_2_ incubator.

### Irradiation

Cells were irradiated at 120 kV by an X-ray machine (ISOVOLT Titan; GE, Ahrensburg, Germany) with IR doses of 2 Gy. Unirradiated samples were processed along with irradiated samples.

### Flow cytometry

Apoptosis and necrosis were detected by the Annexin-V-APC (Annexin-V-Allophycocyanin) and 7AAD (7-Aminoactinomycin) assay (BD Pharmingen, Franklin Lakes, USA). Briefly, cells were suspended in Ringer solution and stained with Annexin-V-APC (dilution 1:40) and 7AAD (dilution 1:40) for 30 min at 4 °C. Cell cycle analysis was performed by Hoechst 33342 staining. Cells were incubated for at least 1 h in ethanol at 4 °C and staining was performed by Hoechst 33342 (60 μmol/l) in Ringer solution. Flow cytometric measurements were performed with Gallios Cytometer (Gallios Cytometer 1.1 Software Beckmann Coulter, Krefeld, Germany) using 25.000 cells per run. Results were analyzed with Kaluza Flow Cytometry Analysis 1.5a (Beckmann Coulter, Krefeld, Germany). Each experiment was performed three times with three replicates per run.

### Immunostaining

SBL5 primary fibroblasts were seeded on cover slips in Quadriperm vessels and allowed to adhere for at least 2 days prior to each Idelalisib treatment. Cells were irradiated as described and fixed for 15 min in 4% paraformaldehyde and permeabilized in 0.1% Triton X-100, blocked in 10% fetal calf serum in Tris buffered saline over night at 4 °C. Cells were incubated for 2 h at room temperature with anti-γH2AX (Upstate, New York, NY) and anti-PML (Santa Cruz Biotechnologies, Dallas, TX, USA). After washing in TBS, cells were incubated with anti-mouse Alexa Fluor 488 and anti-rabbit Alexa Fluor 594 secondary antibodies (Molecular Probes, Karlsruhe, Germany) for 1 h at room temperature. Cells were counterstained with 4′,6-diamidino-2-phenylindole (DAPI) and mounted in Vectashield (Vector Laboratories, Peterborough, UK).

### Fluorescence in situ hybridization

Whole blood sample of the index patient was drawn 3 months after the end of the penultimate radiation therapy. One part of the sample was irradiated with a dose of 2 Gy, the other part stayed mock irradiated. The blood was cultivated and lymphocytes stimulated with Phytohemagglutinin. After repair time of 48 h chromosomes were arrested during metaphase by colcemid and prepared on slides. Chromosome 1, 2 and 4 were stained with chromosome specific probes. Aberrations of at least 100 metaphases were analyzed regarding the amount of double strand breaks. The breaks per metaphase (B/M) value of the irradiated sample was then corrected by the background of the mock irradiated attempt (2 Gy corr.). An increased radiosensitivity is assumed with a resulting value of 0.55 B/M or higher [6–8]. For comparison B/M values from 218 healthy individuals, 452 patients with various cancer diseases and 13 radiosensitive individuals were used.

### Idelalisib concentration series

Whole blood samples were drawn from a healthy 29-year-old Caucasian female. The samples were incubated ex vivo with different concentrations of Idelalisib (no drug, 10 nmol/l and 1 μmol/l) for 4 h. One part of each attempt was then irradiated with 2 Gy, whereas the other part stayed mock irradiated. Lymphocytes were then stimulated, chromosomes prepared and stained as described before. The experiment was repeated for at least three times.

### Statistical methods

Mann-Whitney U Test was used for data analysis.

## Results

### A 73-year-old man with a twelve year history of B-CLL

The initial diagnosis was set in 2003 with a clinical manifestation of generalized lymphadenopathy. After histological diagnosis a treatment with Chlorambucil and monthly immunoglobulin substitution was started. After a long period of clinical regression, a recurrent disease of the right frontoparietal skin was histologically proven in 2012. 6 cycles of R-Bendamustin-scheme were applied and a radiation therapy to the persisting lesion was performed. 36 Gy in 18 fractions were applied and well tolerated. No significant adverse effect to mucosa and the skin were observed. Complete hematologic remission could be maintained until February 2014, when a local lymphoma manifestation of the larynx with stenosis of hypopharynx was histologically proven. Eight cycles of R-CHOP followed and were well tolerated. In November 2014 a recurrence of the right lower leg and shortly later in January 2015 an extensive lymphoma manifestation including multiple both-sided paranasal sinuses (frontal, maxillary and sphenoidal sinuses) as well as the ventral base of the skull, orbital, perioral and frontal infiltration were diagnosed.

**Fig. 1:**
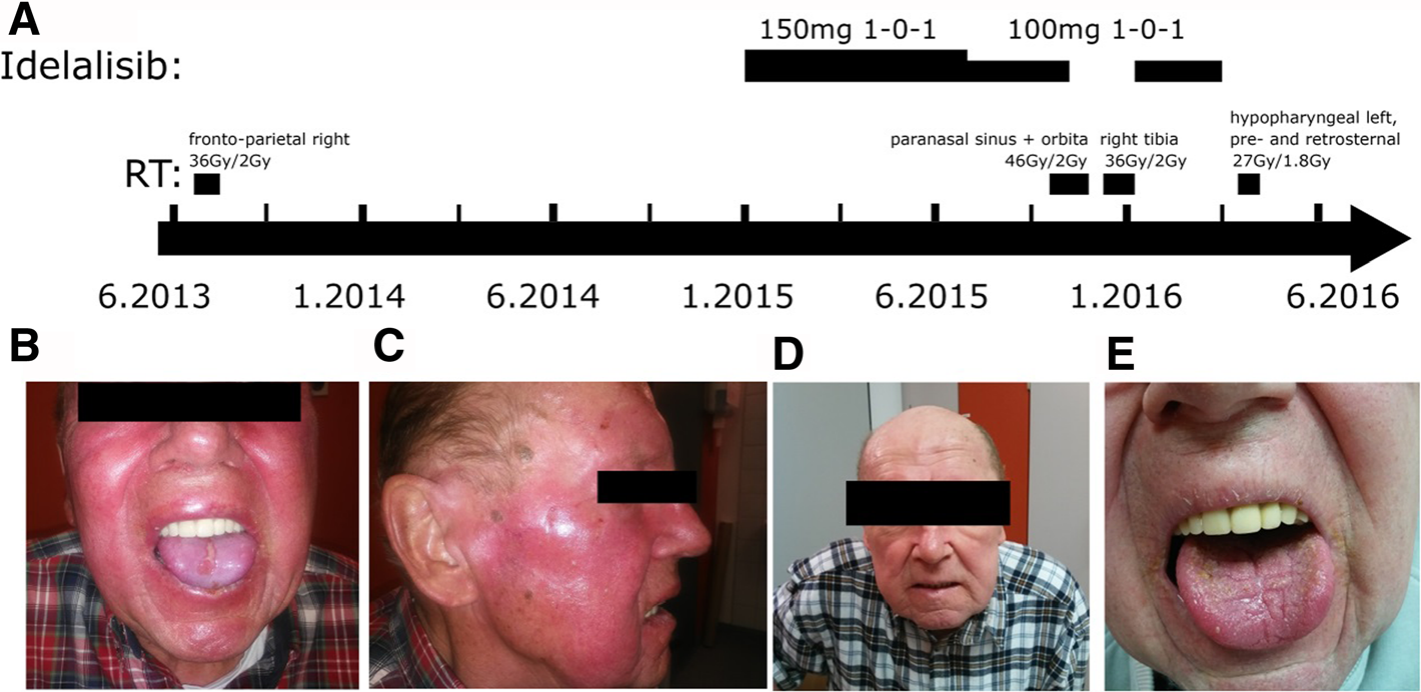
Patients’ side effects due to combined irradiation and Idelalisib therapy. **a** Time line of patients’ treatment with Idelalisib and RT. Patients appearance after 15 days of treatment and a received dose of 24 Gy/2 Gy per day. **b** Enoral mucositis grade 3 and dermatitis grade 2, **c** dermatitis grade 2. **d**, **e** Patients appearance 18 weeks after end of RT

### Patient treatment with Idelalisib

A systemic therapy with 8 courses Rituximab and Idelalisib 150 mg twice daily was started in January 2015 (Fig. 1a). In July 2015 an Idelalisib induced colitis grade 3 with diarrhea frequency of 6 times per day and an exanthema of the face with disseminated plaques on both arms occurred. The patient was internalized and a parenteral rehydration therapy was started. A slightly increased scaling of the face skin had already existed previously. Now the symptoms strongly increased and a parapsoriasis was diagnosed. The patient was treated with topical corticoids, thus the dermal symptoms improved. Idelalisib was paused for a few weeks until full recovery of symptoms and then re-started. On 21 of September 2015 the irradiation of the viscerocranial tumor was started while Idelalisib was continued with 100 mg twice daily. After 20 Gy in 2 Gy fractions a strong radiodermatitis grade 2 and mucositis grade 3 occurred (Fig. 1b, c). Idelalisib was paused and after reaching 26 Gy, radiotherapy (RT) was paused for four days and then continued with a reduced volume stereotactic technique up to 46 Gy. Maximum supportive therapy was needed to finish the radiation therapy. There was no radiation field interference between the frontoparietal radiotherapy in 2013 and the viscerocranial radiotherapy in 2015. The 30% isodose did barely reach the frontal sinus from lateral direction and ended 3 cm above the orbita.

Due to increasing pain in December 2015 radiotherapy with 36 Gy in 18 fractions was applied to the manifestation of the right lower leg. Idelalisib still remained paused during the radiation of the leg. Here a radiodermatitis grade 1 was registered. Idelalisib was re-started in January 2016 with reduced dosing of 100 mg twice daily and was discontinued at the end of March 2016.

In a clinical visit end of February 2016 the patient reported well-being, but a weak general condition. The CT scan showed persisting swelling especially of the right sphenoidal and frontal sinus and the left maxillary sinus. A radiological discrimination between persisting tumor and post therapeutic affection was not possible. Histological confirmation was not initiated. Concerning radiation toxicity, there was no more persistence of radiation dermatitis of the facial skin (Fig. 1d, e). The patient reported neither a taste or smell disorder or dysphagia. Still, he reported moderate hoarseness with a good preserved understanding and an entropion of the right lower eyelid with xerophthalmia and epiphora. Idelalisib medication was stopped in March 2016 due to progressive disease and acute CMV-pneumonia.

In March 2016 radiotherapy was applied to the hypopharynx and retro sternum up to a dose of 27 Gy in 14 fractions. The therapy was interrupted due to a very poor physical condition of the patient; he was transferred to a hospice. There he died in May 2016 as a result of a general progress of the disease.

### Idelalisib and lymphocytes radiosensitivity

**Fig. 2:**
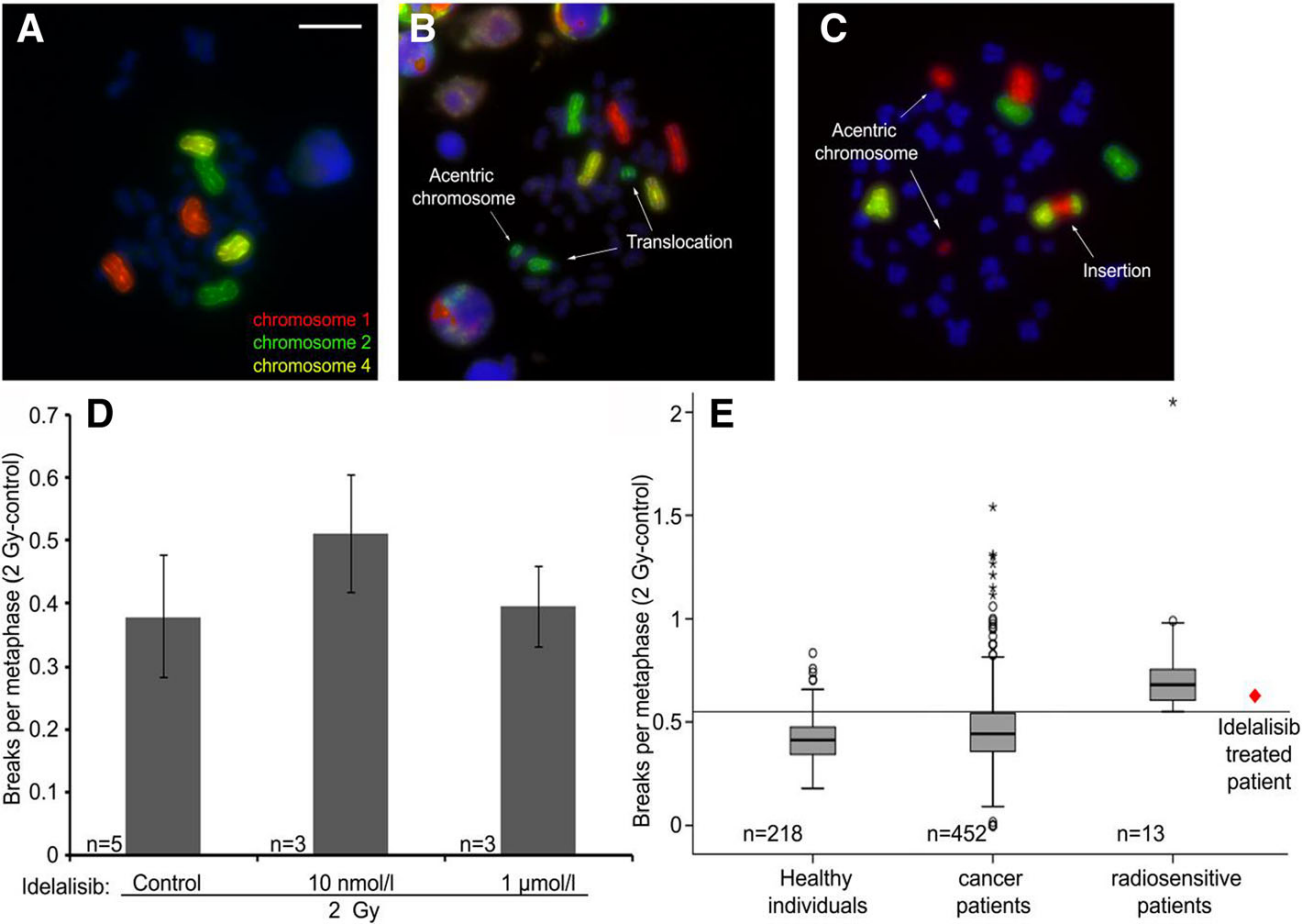
Individual radiosensitivity testing of the patients lymphocytes under Idelalisib compared to different control groups. Three-color FISH painting of chromosomes 1 (*red*), 2 (*green*) and 4 (*yellow*). **a** A metaphase without aberrations, **b** a metaphase with one translocation and one acentric fragment and **c** a metaphase with one insertion and two acentric fragments are displayed. The aberrations were scored as 3 breaks per metaphase (B/M) and 5 (B/M). **﻿d﻿ ** Lymphocytes of a healthy individual were irradiated in vitro with 2 Gy and 10 nmol/l or 1 μmol/l Idelalisib and were compared to a control without Idelalisib treatment. **e** Lymphocytes were irradiated ex vivo with 2 Gy. B/M found in nonirradiated metaphases were subtracted from those scored in the irradiated samples. The patients individual radiosensitivity is compared to groups of healthy controls, cancer patients and radiosensitive patients

Radiosensitivity in lymphocytes was analyzed with a three-color fluorescence in situ hybridization assay (Fig. 2a-c). A combined treatment of lymphocytes with 2 Gy radiation and 10 nmol/l Idelalisib tended to an increased radiosensitivity, while the very high concentration of 1 μmol/l Idelalisib did not (Fig. 2d). The index patient had a radiosensitivity of 0.62 B/M under Idelalisib. Compared to healthy individuals and cancer patients without a treatment with Idelalisib, the value of the index patient is above the third quartile of both groups and is in the range of clearly radiosensitive patients (Fig. 2e).

### Idelalisib and primary fibroblasts radiosensitivity

**Fig. 3:**
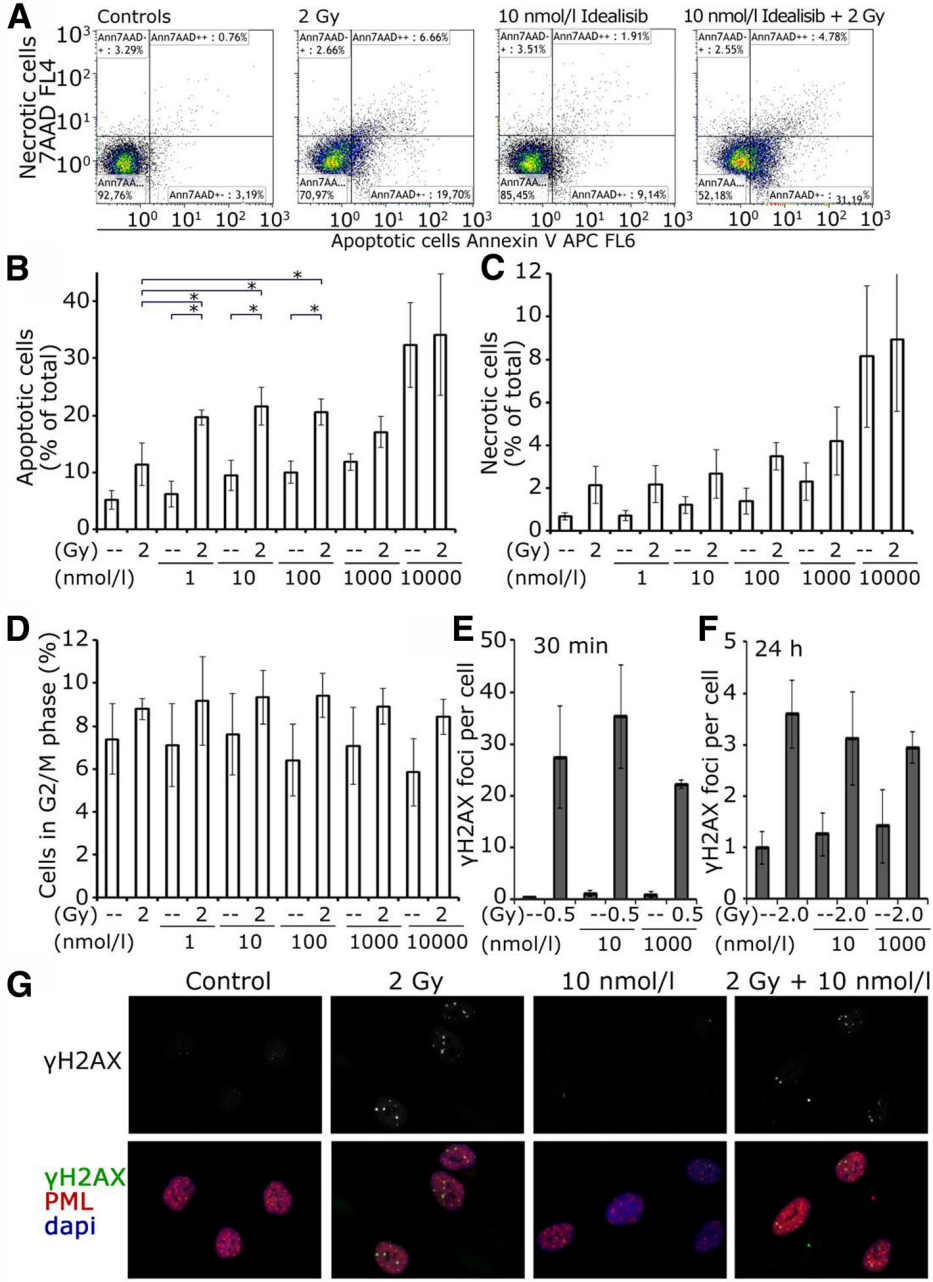
Analysis of cytotoxicity, cell cycle effects and DNA double strand break repair of Idelalisib. Primary skin fibroblasts were treated for 48 h with a concentration of 10 nmol/l of Idelalisib. Apoptosis and necrosis were detected by Annexin V-APC/7AAD staining and flow cytometry. **a** An example of the gating in the FACS plots is shown for untreated, 2 Gy IR, 10 nmol/l and combined treated cells. Induction of **b** apoptosis and **c** necrosis at different Idelalisib concentrations with and w/o irradiation of 2 Gy. Cell cycle phase of primary skin fibroblasts was detected by Hoechst 33342 staining. **d** Induction of G2/M-phase at different Idelalisib concentrations with and w/o irradiation of 2 Gy. DNA double strand breaks were detected by γH2AX immunostaining in primary skin fibroblasts. **e** Initial DNA double strand breaks after 0.5 Gy and 30 min recovery time and **f** after 2 Gy and 24 h recovery time. **g** An example γH2AX, PML-nuclear bodies and nuclear (Dapi) stained cells is given. Significant differences with *p* = 0.05 are marked as *

Primary skin fibroblasts were studied for apoptosis and necrosis using the Annexin V/7AAD approach (Fig. 3a). We found a clear increase of apoptosis at the combined treatment in the Idelalisib dose range of 1 to 100 nmol/l compared to solely irradiated cells or solely Idelalisib treated cells (*p* < 0.05) (Fig. 3b). There is no increase of necrotic cells (Fig. 3c). Likewise, there is no clear change of fibroblasts in the different cell cycle phases: Whether solely irradiated with 2 Gy or a combination of 2 Gy IR and an Idelalisib concentration between 1 nmol/l and 10 μmol/l, the percentage of fibroblasts in the G2/M phase was nearly the same. DNA double strand breaks (dsb) were identified by γH2AX immunostaining. No clear increase of damage was in the initial (Fig. 3e-g) nor in the remaining DSBs.

## Discussion

One patient suffering from B-CLL was treated with four different radiotherapeutic treatments. He experienced distinct increased side effects of grade 2 dermatitis and grade 3 mucositis after a combined RT-Idelalisib treatment. The dermatitis and mucositis already appeared after a 2 Gy fractionated total dose of 20 Gy. There were no side effects caused by one RT prior to Idelalisib treatment and one RT upon completion of Idelalisib. The radiosensitivity of the index patients using the three-color fluorescence in situ hybridization analyzes showed a clear increase of the radiosensitivity level in index patients under Idelalisib. In primary skin fibroblasts a distinct increase of apoptotic rate was found. Summarizing these data, it is a strong indication that Idelalisib can trigger severe acute side effects by RT [9].

A Limitation of this study could be the fact that the patient suffered from parapsoriasis. However, both RTs prior and post Idelalisib treatment were well tolerated with doses higher than the 20 Gy –radiotherapy which caused the grade 2 dermatitis and grade 3 mucositis. Besides, it must be taken under concideration that we measured an increase of radiosensitivity during Idelalisib treatment but the existing rediosensitivity without Idelalisib is unknown as we could not perform the respective testing. Yet the addition of 10 nmol/l Idelalisib to a healthy individuals blood increased the radiosensitivity.

In patients’ blood a concentration of 0.7–10 nmol/l Idelalisib is observed [10]. Therefore it is comparable to the 10 nmol/l of the used concentrations in our experiments. The higher concentration of 1 μmol/l did not increase radiosensitivity. Analogous to that, the apoptosis rate was distinctively increased in the dose range between 1 and 100 nmol/l Idelalisib, whereas the increase was less clear using the high concentration of 1 μmol/l.

It is noteworthy that combined treatment of IR and Idelalisib induces a selective induction of apoptosis in fibroblasts. There is only very limited influence on DNA dsb-induction or repair, on cell cycle and on necrosis. This hints to influence of Idelalisib on apoptosis through a specific pathway. Idelalisib inhibits in B-cells the p110δ catalytic subunit of PI3K-δ very specificly [11]. This enzyme is primarily expressed in the hematopoietic system [12]. It induces apoptosis specifically in malignant B-cells [13]. No significant off-target activity exists at a concentration of 10 nmol/l [13]. There is no effect on DNA dsb, cell cycle or cytotoxicity in fibroblasts up to 1 μmol/l. However, in combination with ionizing radiation a clearly specific induction of apoptosis exists and it hints on an off-target activity which is in the DNA-damage signal transduction pathway, leading to a nearly exclusive induction of apoptosis.

If a combined treatment of Idelalisib and IR is indicated, the high risk of side effects should be considered thoroughly. The main reason for concomitant treatment is the concern for an intensified tumor growth during the time, when the drug is omitted. Yet the acute side effects were manageable. Patients under treatment with Idelalisib should be monitored more particularly to detect and treat toxicities early enough. They should be provided with supportive care similar to patients receiving simultaneous radiotherapy and a BRAF inhibitor or other multikinase inhibitors [14–16]. Nevertheless, the results of this analysis show the feasibility of RT with concomitant Idelalisib therapy. Like the radio sensitizing effect we described on fibroblasts in our study, Idelalisib might sensitize cancer cells analogically by induction of apoptosis. This would be a favoring argument for a concomitant treatment and could lead to improved tumor control.

## Conclusion

Idelalisib is radio sensitizing. In combination with radiation it clearly shows a specific induction of apoptosis in cell cultures. A combined Idelalisib radiotherapy treatment has an increased risk of side effects. Facing them, a pause of Idelalisib medication during radiotherapy should be taken into consideration. However, combined therapy seems to be feasible when patients are monitored closely during and after radiotherapy.

## Abbreviations

7AAD: 7-Aminoactinomycin D
Anti-PML: Anti- Promyelocytic leukemia protein
BCR: B-cell receptor
CLL: Chronic lymphocytic leucaemia
CMV: Cytomegalovirus
CXCR4: C-X-C chemokine receptor type 4
Dsb: Double strand breaks
Gy: Gray
IR: Irradiation
mTOR: Mechanistic Target of Rapamycin
PI3K-δ: Phosphatidylinositol 3-kinase-δ
RPMI culture: Roswell Park Memorial Institute culture
RT: Radiotherapy
TBS: Tris-buffered saline
